# Supplementary material for: Identification of promoter activity in gene-less cassettes from Vibrionaceae superintegrons
Source: Nucleic Acids Res. 2024 Jan 12;52(6):2961–76. doi: 10.1093/nar/gkad1252 (PMC11014356; doi:10.1093/nar/gkad1252)
Supplement: gkad1252_Supplemental_File [file gkad1252_supplemental_file.pdf]

Supplementary Table S1. Fragments sequences cloned in pDProm

Name	Origin species	Sequence (5' → 3')
mCherry	-	GCAGTGAGCGCAACGCAATTTTACTTGTACAGCTCGTCCATGCCGCCGGTGGAGTGGCGGCCCTCGGCGC<br>GTTCGTACTGTTCCACGATGGTGTAGTCCTCGTTGTGGGAGGTGATGTCCAACTTGATGTTGACGTTGTAG<br>GCGCCGGGCAGCTGCACGGGCTTCTTGGCCTTGTAGGTGGTCTTGACCTCAGCGTCGTAGTGGCCGCCGT<br>CCTTCAGCTTCAGCCTCTGCTTGATCTCGCCCTTCAGGGCGCCGTCCTCGGGGTACATCCGCTCGGAGGAG<br>GCCTCCCAGCCCATGGTCTTCTTCTGCATTACGGGGCCGTCGGAGGGGAAGTTGGTGCCGCGCAGCTTCA<br>CCTTGTAGATGAACTCGCCGTCTTGCAGGGAGGAGTCCTGGGTCACGGTCACCACGCCGCCGTCCTCGAA<br>GTTCATCACGCGCTCCCACTTGAAGCCCTCGGGGAAGGACAGCTTCAAGTAGTCGGGGATGTCGGCGGG<br>GTGCTTCACGTAGGCCTTGGAGCCGTACATGAACTGAGGGGACAGGATGTCCCAGGCGAAGGGCAGGGG<br>GCCACCCTTGGTCACCTTCAGCTTGGCGGTCTGGGTGCCCTCGTAGGGGCGGCCCTCGCCCTCGCCCTCG<br>ATCTCGAACTCGTGGCCGTTCACGGAGCCCTCCATGTGCACCTTGAAGCGCATGAACTCCTTGATGATGG<br>CCATGTTATCCTCCTCGCCCTTGCTCACCATGGCTTGCTCCTTAGGAGCAAGCCATGAGTAAAGG
P_{lac} *attC gfp*	-	CTCACCATGGCTTGCTCCTTAATGTGAGTTAGCTCACTCATTAGGCACCCCAGGCTTTACACTTTATGCTT<br>CCGGCTCGTATGTTGTGTGGAATTGTGAGCGGATAACAATTTCACACAGGAAACAGCTATGAGTTAACAA<br>ACGCCTCAAGAGGGACTGTCAACGCGTGGCGTTTCCAGTCCCATTGAGCCGCGGTGGTTACGGTTGGTGT<br>GTTTGAGTTTTGTGTTATGCGTTGTCAGCCCCTTAGGCGGGCGTTAGCCAGGAGCAAGCCATGAGTAAAG<br>G
P_{lac} *attC mCherry*	-	CTCACCATGGCTTGCTCCTTGGCTAACGCCCGCCTAAGGGGCTGACAACGCATAACACAAAACTCAAACA<br>CACCAACCGTAACCACCGCGGCTCAATGGGACTGGAAACGCCACGCGTTGACAGTCCCTCTTGAGGCGTT<br>TGTTAACTCATAGCTGTTTCCTGTGTGAAATTGTTATCCGCTCACAATTCCACACAACATACGAGCCGGAA<br>GCATAAAGTGTAAAGCCTGGGGTGCCTAATGAGTGAGCTAACTCACATTAGGAGCAAGCCATGAGTAAA<br>GG
C1	*V. cholerae* N16961	CTCACCATGGCTTGCTCCTTTTAGTTGCCAACGAGAAAAAAGTACCTAAAGCAGAATACTTAGGGCTAAT<br>GTCCGTGTGTTTTTGGCTAGGTTTTCGTATTTCAATTTCTAGTTATTAGGTGTTGTGAAAATTGTTCTTTGG<br>TTCCTTGCGCGATTTGGGCTTTCTGTTTCAGAGGCTGTTAGCTTGTTGAAAGCCTGCTCTGCTGCATTGTC<br>GTTCCAAAAGGTTTGTTTGGTGTTCAAAGTCCGTTTTCTACGGCGTTGTAAATTCCAAGTGGTTTCAGTGA<br>CAGGCGCTTTGAAACTGCGCCTGATTTGAGTTTCTTAATACACGTAAAGCTCGTGTTGGCAAAGCGGCAA<br>TCCACTTCAAATGAAAGTGTTAGGTCTGGCAACTAACAAAGCATTCAAGAGGGATTCACAACGCTTGGCA<br>GTTTTGGTTTGAATCAGCTTTAGTGTTTACTGCACAATAGCTTAGGTTGGGTGGTGGCGTTGTTCACCCCT<br>TAATGCGGCGTTATGTAGGAGCAAGCCATGAGTAAAGG

C2	*V. cholerae* N16961	CTCACCATGGCTTGCTCCTTTTATGCTTAATCCATAAAAATCAGCAGGTTGTAATTGTCGATTTCTTTGGC<br>ATTTCATTCAGGTTTTGTCGGCAATTCAATATTGTTTTTCGCTGCCTAATGAAGCGGCAATGTATCTGGTG<br>CTGAAAATTCAGTGTTATTGCCTCATTGAGTTGTCGCGCCCATGCGTGGTGAGTGAGTTTGTCGGAGAGG<br>TTTTCACATTGGCCTAGTTGCTAAATGAAAGCCTGCATTGGTCAGTTCATAAGTAAAAGTTGAGCCAGTT<br>CTAATTCAAGGAAATTCGATGTTTGGTCAATAATTTCAGTGAGTTACTCTTGAGTTGGCTCAAATGTAAA<br>GTGTTGAAGCTTAAGCATAACAAACGCCTCAAGAGGGACTGTCAACGCGTGGCGTTTCCAGTCCCATTGA<br>GCCGCGGTGGTTGCAGTTGTTGTGTTTGAGTTTAGTGTTATGCGTTGTCAGCCCCTTAGGCGGGCGTTATG<br>TAGGAGCAAGCCATGAGTAAAGG
C3	*V. cholerae* N16961	CTCACCATGGCTTGCTCCTTTTAGTTTCTCTCAGGAAAATCATCAAAAATTCACGCACAAGGTTCTGAAAA<br>TTCAGCCTTTATCGTTCAAGCTGCACAATTTGGTTTTGGGGTTTTTCCTGATGTTGATTTGGTAGAAAGCG<br>TTGAAAGTTCAGCGGTTTCAAAACCGCGGATAACCAACAAGCTTTGATGCTTCAATGTTGTCGGACGTTC<br>AACTTGATCAGTTTGGTTTCATAAAAGGTTGAATCATCGCTGTGATCTGGCTTTCTTTGTGCGGACTCTTA<br>ACGTTTTGGCAGCCAACTTCACAGCTTTTGGCGTTGGACGTGTGTTTGCGTGATGCTTTTGCGTAAAATGA<br>CTTTCAAGAAAATGAGTTTAAAAAGTCATTGTTAAACAATAGGCTACGAAACTAACAAACGCCTCAAAG<br>GGACTGCCAACGCGTGGCGTTTCCAGTCCCATTGAGCCGCAGTGGTTTCGGTTGTTGTGTTTGAGTTTTGT<br>GTAATGCGTTGTCAGCCCCTTAGGCGGGCGTTATGTAGGAGCAAGCCATGAGTAAAGG
C4	*V. cholerae* N16961	CTCACCATGGCTTGCTCCTTTTAGCTTCTTGAAGCAAAAATTATCAAAGTCTGGATTTAATTGTCCTGAAA<br>ATTCAGCTTTTAGTGTTCAAGTTTCACAATTTGGCTTTCGAGTCTTTCTGATGCTTATTTGGTAGAAAGTGT<br>GGAATCTTCAGCAGTTTCAAAGCCGCTAAAAATCATCAAACCTTGATGCTTCAATGTTGTTGGATGCTCC<br>AAACGGAAAGCTTGGTTTCACAAAGGCTGCATCATCGCTGAAAGAGTGTTTTCTTTGTCACGGACTCTTA<br>ACGTGGCCAACTTAACCTTGAATGCTTTGAGTTTTGGCTGTTTACTTCACAGCTTTTGGTGCTGGACGTGC<br>GTTTGTTCGATGCTTTTGCGTAAAATTGCTTTCTCAAAATCATTGTTAAACAATGGGCTACGAAGCTAACA<br>AACGCCTCAAGCGGGACTGTCAACGCGTGGCGTTTCCAGTCCCATTGAGCCGCGGTGGTTTCGATTGTTG<br>AGGTTGAGTTTAGTGTTAATGCGTTGCCAGCCCCTTAGGCGGGCGTTATGTAGGAGCAAGCCATGAGTAA<br>AGG
C5	*V. cholerae* N16961	CTCACCATGGCTTGCTCCTTTTAGCTTCTTAAAGCAAAAATCGGTTTAATTGTCCTAAAAATTCAGATTTT<br>AGCGTTCAAATTGCACAATTTGGCTTTTGAGCTTTTCTTGATGCTGATTTGGTCGAACGTGTTGAAAGTTC<br>AGTGGGTGCAAAGTCGCTGAAAACTAGTAAGCCTCGATGCTTCAACGTTGTTGGATGTTCCGCTCGGAAA<br>GTTTGATTTCACAAAATGCTGCATCATCGCTGTGATCTTGCTTTCTTTGTCGCGGACTCTTAACCGTGGCC<br>AACTTAACCTTGATCGTTTTAAGTTTTGGTAGCCAACGTCACAGCTTTTGGCGTTGGACGTGTGCTTGCTT<br>GATTCTTTTGCGTAAAATGCCTTTCAAACAAATGTGTTAAAAGCCATTGTTAAACAATAGGCTACGAAGC<br>TAACAAACGCCTCAAGAGGGACTGTCAACGCGTGGCGTTTCCAGTCCCAATGAGCCGCGGTGGTTACGGT

		TGTTGTGTTTGGGTTTAGTAGTAATGCGTTGCCAGCCCCTTAGGCGGGCGTTATGTAGGAGCAAGCCATGAGTAAAGG
C6	*V. cholerae* N16961	CTCACCATGGCTTGCTCCTTTTAGCTTCTTAAAGGCTAAGATCATCAAAATCCAAACTTCTTGTTCTGAAAGATCAGTTTTTAGTGTTCAAAGTGCACAATTTGGCATTTCAATTTTGCTAGATGCTGATTTGGTAGAAAGTGTTGGAAGTTCCGCAGTTTCAAAGCCGCTGAAAGCCAACAAGTCTCGATGCTTCAAAGTTGTTGAATGTTCAATTCGGTCAACTTGGTTTCACAAAAAGCAACATCGTCGCTGAAATCGTGCTTCTTTGTCGGAAATGCAGTAAGTGGCCAACTTAGCCTTGAACGCTGCAAGTTTTGGCAGCTCACTTCACAGCTTTTGGCGTTGGACGTGTGTTTGAGTGATGCTTTTGCGTAAAATGCATTTCAAGCAAATGTGTTTAAAAAGTCATTGGTAAACAATGGGCTACGAAACTAACAAACGCCTCAAGAGGGACTGTCAACGCGTAGCGTTTCCAGTCCCAATGAGCCGTAGTGGTTACGGTTGTTGTGTTTGAGTTTGGTGGAAATGCGTTGCCAGCCCCTTAGGCGGGCGTTATGTAGGAGCAAGCCATGAGTAAAGG
C7	*V. cholerae* N16961	CTCACCATGGCTTGCTCCTTTTATGCTTAATCCATAAAAATCAGCAGGTTGTGAATGTCGATTCCTTTGGGATTTCGTTCAGGTTTTGTCGGCAATTCAATATTGTTTGTCGCTGCCTAATGAAGCTGCAATGTGTCTGGCGCTGTAAATTCTGAGTTGTTGCTTCACTAGGTTTTTGAGTCTGCGCGAGGTTAGTCTTGTTGGCTCAAGTCGCTTTGAGCAGTCTGGTGCCTTTACTTACAGGCTTAAAGTCTGTCGGCAATTGAATATTGTTTTGCGCTGCCTAATGAAACAGCAATGTGTCTGTCGCTGTAAATTTCAGTGTTATTGCCTCATTGAGTTGTCGCGCCCATGCGTGGCGAGTGAGTTTGTCGGAGAGGGTTTCCACATTGGCCTAGTTGCTAAATGAAAGCCTGCATTGGTCAGTTCATAGGTGAAATTTTAGCCAGTTCTAATTCAAGGAAACTCGGAATTTGGCCAATGATTTCATTGGGTTACTCTTGATTTGGTTCAAATGTAAAGTGTTGAAGCTTAAGCATAACAAACGCCTCAAGAGGGACTGTCAACGCGTAGCGTTTCCAGTCCCATTGAGCCGCGGTGGTTACGGTTGTTGTGTTTGAGTTTGGTGTTATGCGTTGTCAGCCCCTTAGGCGGGCGTTATGTAGGAGCAAGCCATGAGTAAAGG
C8	*V. cholerae* N16961	CTCACCATGGCTTGCTCCTTTTAGCTTCTTAAAGCAAAAATCATCAAAATCTGGGTTTAATTGTCCTAAAAATTCAGATTTTAGCGTTCAAATTGCACAATTTGGCTTTTGAGCTTTTCTTGATGCTGATTTGGTCGAACGTGTTGAAAGTTCAGTGGGTGCAAAGTCGCTGAAAACTAGTAAGCCTCGATGCTTCAACGTTGTTGGATGTTCCGCTCGGAAAGTTTGATTTCACAAAATGCTGCATCATCGCTGTGATCTTGCTTTCTTTGTCGCGGACTCTTAACCGTGGCCAACTTAACCTTGATCGTTTTAAGTTTTGGTAGCCAACGTCACAGCTTTTGGCGTTGGACGTGTGCTTGCTTGATTCTTTTGCGTAAAATGCCTTTCAAACAAATGTGTTAAAAGCCATTGTTAAACAATAGGCTACGAAGCTAACAAACGCCTCAAGAGGGACTGTCAACGCGTGGCGTTTCCAGTCCCAATGAGCCGCGGTGGTTACGGTTGTTGTGTTTGGGTTTAGTAGTAATGCGTTGCCAGCCCCTTAGGCGGGCGTTATGTAGGAGCAAGCCATGAGTAAAGG
C9	*V. cholerae* N16961	CTCACCATGGCTTGCTCCTTTTAGCTTCTTAAAGGCTAAAATCATCAAAATCCCAACTTCTTGTTCTGAAAAATCAGCTTTTAGTATTCAAATTGCACAATTTGGCATTTGAATTTTTCTAGATGCTGATTTGGTAGAAAGT

		GTTGGAAGTTCCGCAGTTTCAAAGCCGCTGAAAGCCAACAAGCCTCGATGCTTCAAAGTTGTTGGATGTT<br>CAACTCGGTCAACTTGGTTTCACAAAAAGCAGCATTGTCGCTGAAATCGCGCTTTCTTTGTCGGAAATTC<br>GGTAAGTGGCCAACTTAACCTTGATCGCTGCGAGTTTTGGCAGCTCACTTCACAGCTTTTGGCGTTAGAC<br>GTGTGTTTGAGTGATGCTTTTGCGTAAAATGCATTTCAAGCAAATGTGTTTAAAAAGTCATTTTTAAACAA<br>TGGGTTATGAAGCTAACAAACGCCTCAAGAGGGACTGTCAACGCGTGGCGTTTCCAGTCCCATTGAGCCG<br>CGGTGGTTTCGGTTGTTGTGTTTGGGTTTGGTTGTTATGCGTTGTCAGCCCCTTAGGCGGGCGTTATGTAG<br>GAGCAAGCCATGAGTAAAGG
C10	*V. cholerae* N16961	CTCACCATGGCTTGCTCCTTTTAGTTTCTCTCAATCAAAAATCATCAAAATCTAAGCTCATTTGCATTAAA<br>CATGCAGCTTTTAGTATCCAAATTTCACAAGTTGGCTTTTGAGTTTTGCTTGATGCTGATTTGGCAGAAAG<br>CGTTGAAAACGCAGTAGTTTCAAAGTCGCCGCAAGCCAACAAGTCAAGATGCTTCAACGTTGTTGGGTGT<br>TCAGCGCGGTCAGCTTAGTCTCACAAAAGGCAGCATCATCGCTGTGATCTGGTTTTCTTTGTCGCGGACTA<br>TTAACAGTGGCCAACTTAACCTTGATCGTTTTAAGTTTTGGTAGCTTGCTTCACAGCTTTTGGTGTTGGAC<br>GTGTGCTTGTAAGATGCATTTGCGTAAAATGCTTTTCAAGTAAAGGTGTTAAAAAACCATTGGTAAACAA<br>TGGGCTACGAAACTAACAAACGCCTCAAGAGGGACTGTCAACGCGTGGCGTTTCCAGTCCCATTGAGCC<br>GCGGTGGTTATGGTTGTTGTGTTTGAGTTTAGTGGTAGTGCGTTGCCAGCCCCTTAGGCGGGCGTTATGTA<br>GGAGCAAGCCATGAGTAAAGG
F1	*V. fischeri* ES114	CTCACCATGGCTTGCTCCTTTTATACGAAATCAATGACGTCCAAATTATAAATATTTAATAGTAACCTACC<br>TGATTTTACAGTTAGTAAGGCAGTGTCAATGTAATAAAATCGTAGTCACTCCTTACTTATGTGTTGCTGTA<br>GACAAGCATTTCATGTCTGGGACTCAATTTTAGCCAATGAGTCCCTTTTTTTTGTGCCGAGGGGGGTAGCG<br>TTAAAAACAAACCTGTCTTATTTTACAGTTGGTTAAGAGTGATTCCAGTAATAAACTGGAGCTCCTCATA<br>ATTTTTGTGCTGCTGTAGACAGGCATTTCACGTTCAGGGTTCGATTCCAGTCATCGAGCCCTTTTTTTATTC<br>ATATCTCTTGTGTTGTTTTAGCTGTGATAAGATGTAATTTATATTAAGTTTTGTATATATGCTTATTCAAAC<br>TAAAGTAGACACGTATAACAAATGCATCAACACGATTTGCTACATTCGGCATTCTAGATTTCTTTGGGTTT<br>GCCGCGTTAAGTGGTAAATTTAAGCTTAATCTGCATGGTAGCAAACGTGTTATGCAGGTGTTATGTAGGA<br>GCAAGCCATGAGTAAAGG
F2	*V. fischeri* ES114	CTCACCATGGCTTGCTCCTTTTATACGAACTCTGAGAATGCAGTTTTTTGGTTCAGTTCAGCGATTGAACC<br>ATTAGTTTTTTAGGTGTGAAAGTAAGGTGGTTTTCCTTTTTCTTTGTTGCCTTTTAGTGTTCGTTTCATCAG<br>GTTCGGCATTGAACTATTATTGCCCCAAATTCAGGCGTTTCAGAAACTAATTTACGCTTCAATTGTTCATG<br>GTTTTGCCTCATTTTAGCCTGCATCAGTGTTTTCCTCGCAGCATTTTCAATCCCAATTGTGGTTAGGATATT<br>TGGTTTATTAGCTCAGAAGTTGATGAAACCAAATCGTACAAATAGTGTAAGAAAAAGGGATTGTTTCGTT<br>TCACAATTATTGTTGTTGGTTGGATATTTTGCTCGCTTAATTGAGCCAGTCTAATTAAACTCAGTTTACAG<br>CAAATCTTGGTAAATCAGTTCTTTACCAAGTTAATTGGAGCGAAAGCAAGGCTCGTATAACAAAGCAATC

		AACACGATGCTATTTACATTCGGCATTCGCGGTTTGGAGTATAATTGGTTTTGTAAGTCAGTCTTTCGCAC<br>GTGTTATTGTGGCGTTATGTAGGAGCAAGCCATGAGTAAAGG
F3	*V. fischeri* ES114	CTCACCATGGCTTGCTCCTTTTGTGCGTACTAAGCCCGAAATTTTTTGCACGGTTCCTCAATAGAAACGAG<br>ACTTTTTTTAAACGCTATTTAGCTATCACAAATCCTCTTCTTTGTTGCCTTTTAGCGTTCGTTTCATCAGGT<br>TCGGCAATTAAGCATTGTCGGTCTAAGTTCAGGCGTTTCAGAGGCATTTTTACGCTTCAATGGTTCTTTGG<br>TGTTGCATCATTTTACTCTGCATCCGTGTTTTCCTCGCAGCATTTTCAATCCCAGTTGTGGCGAGGATATTT<br>GGTTTATCAGCTCAAAATCTGCTGAAACCAAGTCGTTCAAATGGTGTAAAAAATGTATCTGTTTTCCATTT<br>ACTTCATCTCGCAGTTTAAGAACATTTTGTTCGCTTAATTAGCATCGTTAAATTAGAATCAGGGCTTAGAC<br>AATTTTGGCGGGGCAGTTCTTTACCAAGTTGTCCAAGCCGAAAGAGAGTCCGCACAACAATCGCATCAAC<br>ACGATTTACTACACTCGGCAATCTCAGTTTGCCGAGTGTTTCTCGTTTTAGGGCATCAATATTCAGTATAA<br>TTGTATAGTAGTAAACGTGTTATGCAGGCGTTATGTAGGAGCAAGCCATGAGTAAAGG
P1	*V. parahaemolyticus* VPD14	CTCACCATGGCTTGCTCCTTTTAGGCGACGGGCAGGAAAGTTCTTTGTTCTCATCTTCTTAGTTCTCTTTGA<br>GCATTCGTTTTTCTAAGTCGGCAACTTGGTATTGTCGGCTCAAATTCTTGAATCTCTCCAGCCGTAAAACA<br>TGCCAAATTTCGCGGGTTGCCTCATTGGTTTTCAGTGTTGGATGGTGGTGAGTTTGACTTGCTCAAAGTGT<br>GGCAAGGGCAAGTCAATTGAGGCTTTCGATTGCGCTGCTAGTTTGTCGCGTTGGCATTTTCGGGTTCAAA<br>ACCCGCGTTGTTGTTGAAGGTCGGCATCTCGTGGTCGTTGGAATTTTGGCTGAAACATAGTGAGTTTTGTG<br>GTTCTTGAATCACAAATAATCAGTGTATGTTTGGTTGCTTATTTTGTTTCGGGTAATCTGCTTTCAAACAA<br>CCTTGAAACTGGTGTGAAACTTCGCCTAACAAGGCGTTCAAGACGGATTCACAACGCTTGGCGCTCTCGG<br>TTTTCTTTGAGTTAAGTGATTATGTCACAATGGTTTAGGTAGGGTGGTAGGCGTTGCTCACCACTTAACGC<br>GGCGTTATGTAGGAGCAAGCCATGAGTAAAGG
P2	*V. parahaemolyticus* VPD14	CTCACCATGGCTTGCTCCTTTTAGTTGCCAACGAGGAAAACGTACCCAAAGCAGATTGTTTAGGGCTAAT<br>GTCCGTGTGTTTTTGGCTAGGTTTTCGTATTTCAATTTCTAGTTATTTAGGTTTGTGAAAATCGATCGTTGG<br>TTTCTCTTGTGGTTGAATTTTCTATTTCAGAAACCGATTTATATGTTGAAAGTTAGCTGTGTAACATTGTCT<br>GTCCAAAGGAGCTTTTTGGTGTTGTTAGCCCGTTTTCTACGGCGTTGTATGTACCAAGTGGTTTCAGTTAC<br>AGTCGCTTTGAATCTGCGCCTGATTTGAGTTTTTCAACGTACGTAAAGTTAGCGTTGGCAAAGTGGCAATT<br>AACTTCAAATCAAAGTGTTAGACTTGGCAACTAACAAAGCATTTAAGAGGGATTCACAACGCTTGGCGGT<br>TTTGGTTTGAATCGGCTTTAGTGTTTACGGCACAATGGTTTAGGTCAGGTGGTGGCGTTGTTCACCCCTTA<br>ATGCGGCGTTATGTAGGAGCAAGCCATGAGTAAAGG
P3	*V. parahaemolyticus* VPD14	CTCACCATGGCTTGCTCCTTTTAGTCGCCAGAAAAAGCTAGAAGTCAAAATCATGTTTCTAAGCTCAAAA<br>CTTGGCTACTGGTTGTTTGATTCATAGTTCCATCTTCGACGGTCAAAGCTCAGTTTCATCGGTTCTAAATC<br>AATGCAATGCTATTTTCCAAGCGGTTGTTTCGTTCTTTGGTGGTGATAGAGCGGGGCAATCGAAAATCTG<br>TTTTTGGTTGCAAATCTGCTCGGTGAGTTGGTTGGTTTAAAAGCTTATTTACTTGCTGAAATGACGTTTTCT

		CTGGTGTTGTAAGTTCCACGTGGTTTCAGTGACTTTGTTGAAAGTCCGCACTGTTAGATTGGTTTTCCAAA<br>AGCGCTTTTTGGTGTTGTTAGCCCGTTTTCTGCGGCGCTGTTTTTTCTAAGTGGTTTCATTGAGTAGCCGCT<br>TTGAGACTACGCCTGACTTAAGTGCATCAAAACACATAAAGTTTGTTGTTTGCAAAGTTTAGTTCGGCTTA<br>AAATTGTCGCTCGGTGCCTCGCGACTAACAAACTGCTCAAGAGGGATTCGCAACGCTTGGCATTTTTACT<br>ATGCGTTGAATTTAGTGATTAAGGTGGTATGCGGCGGCTTCGGTATTGCGTTGCTCACCCCTTAGCAGGG<br>CGTTATGTAGGAGCAAGCCATGAGTAAAGG
P4	*V. parahaemolyticus* VPD14	CTCACCATGGCTTGCTCCTTTTAGGCGACAGGCAGGAAAGTTCTTTGTTCTTACCTTCTTGTTCCCTCTGGC<br>AATTCGCTTTTCGTAGTCGGCAATTAAGCATTGTCGGCTCAACTTCTTGAATCTCTCCAGCCGTAAAACAT<br>GCAAAGGTTCGTGGGTTGCCTCATTAGTTTTCATTGTCGGTTGGTGGATAATTTCACTGGCTCAAAGTGTG<br>GCAGGGGCAAGTTAATCTCGGCTTTCGTTCTCGCTGTAAGTTTGTAGCTTTACGTTTCGCTTGCTCAAAAG<br>CAACTTGAACGCTGAAAGTCAGCATTTTGTGGTTGTGGATATATGGGCTGAAACATAGTGGCTTTTGTGG<br>TTCTTGAACCACAAAAAGTCAGTGGGTGTTTGTTGCTTATAAGGTTTCGGGTAATCTATTTTCAAACCAAC<br>TTAAACACTGGTGGGAAACTTCGCCTAACAAGGCGTTTAAGGCGGATTCACAACGCGTGGCGATTTCAGT<br>TCAAGTCTAGTTTAGTGTTTAAGGTGTAGTGTTTAGGTGTGGTGGTTTGCGTTGTTCACCACTTAACGCGG<br>CGTTATGTAGGAGCAAGCCATGAGTAAAGG
V1	*V. vulnificus* FDAARGOS_663	CTCACCATGGCTTGCTCCTTTTATGCTTAATCAGGTAAAATCAGTGGTTTATGGTTTTTCTTTGCTCCCTCA<br>GCTTTTCAGTTTGGTGTTTGTCGGCAAGTTGGCTGTTTTGAGCGCTGTTTTTCGGACATCCATTCTTTGGCG<br>CTGGAAATTCCGAGAGTTGCCTCAATCAATTTTTGGGTAAGCGCGAGGTTAGGGCGGTTGGCTCAATCAG<br>AAATCCATTTATAGGTTTTCAAACTTCAAACCAGATTTGCCAAAATTCCTAGTCTGATTATCAAAACTATG<br>ATTCATTTCGGTTTTCTACGTTTTGGTTTTTGTTTGTAAATCAAAGTCGAGTTAATCTTGTTTTTAGTAAAA<br>CGTAAGCCATTGAAGCTTAAGCATAACAAGGCGTTTAAGTGGGATTCATGCCGCGTGGCATTTTGGGTAT<br>GCGGTGAATTTTGGTGGTGAAAGTGGTCTGCGGAAAGTTGGTTTAGGCGGCACTCACCCCTTAACGCGGC<br>GTTATGTAGGAGCAAGCCATGAGTAAAGG
V2	*V. vulnificus* FDAARGOS_663	CTCACCATGGCTTGCTCCTTTTATGTTTAGTCACGTAAAATCAGTTGGTTGCATCTTTTCTTTGCTTTCTCA<br>GCTTTCTAATTTTGGTCTTGTCAGCAAGTTAACTTCAGTGTTCGCTGTTTTCTGGACTCCAATTCGTGGGCG<br>CGAAAAATTCAGAGAATTGCCTCATTGAAATTCAGTGCGCTTGTGAGTCGAATGAAGCTGCCTTAGTTTT<br>AAGTTTGAGCTCAAGTTTTCTGTTTCTCACGCTAAAACCCAAAGTCATTAAAACTAAATTTATGAGTTAAT<br>TCAGCTTTTTAGCGTCTGGTTTTGGTTTGCAAAGGTAAGTCGAGTTAAGCTCTTGGTATCGCAAACTCAAC<br>CCTTTGTGTCTTAAACATAACAAGGCGTTTAAGCGGGATTCATGCCGCGTGGCATTTTTGGTTTGTAGTGA<br>GTTTTGGTGGTGAAAGTGGTCTGCGGCAGCTTGGTTTATGCGGCATTCACCCCTTAACGCAGCGTTATGTA<br>GGAGCAAGCCATGAGTAAAGG

V3	*V. vulnificus* FDAARGOS_663	CTCACCATGGCTTGCTCCTTTTATGTTTAGTCACGTAAAATCAGTTGGTTGCATCTTTTCTTTGTACTCTCG<br>GCTTTCTAATTTTGGTCTTGTCGGCAGGTTAACTTCAGTGTTCGCTGTTTTCTGGACTCCAATTCGTGGGCG<br>CTAAAAATTCAGAGAGTTGCCTCATTAAAGTTCAGTGCGTTTTTGAGTCGAATGAAGCGATCTTAGTTTC<br>AAAGTTTGAGCACCAGTTTTTCGGTTTCTCACGCTAAAACTCAATATCAGTACAATCAAATTTATGAGTTA<br>ATTCAGTTTTTTAGCTTCTAGTTTTGGTTTGCAAAGTTAAGTCGAGTTAAGCTATTGGTTTCGTAAAACTT<br>AACCCTTTGTGTCTTAAACATAACAAGGCGTTTAAGAGGGATTCATGCCGCGTGGCATTTTTGGTATGCG<br>GTTGGTTTTGGTGGTGAAAGTGGTCTGCGGAAGGTTCGTTTATGCGGCATTCACCCCTTAACGCGGCGTT<br>ATGTAGGAGCAAGCCATGAGTAAAGG
V4	*V. vulnificus* FDAARGOS_663	CTCACCATGGCTTGCTCCTTTTATGCTTAATCACGTAAAATCAGTGGTTTATGGTTTTTCTTTGTTCCCTAG<br>GCTTTTCAGTTTTGTGTTTGTCGGCAAGCCAGCTTTTTTGAGCACTGTTTTTCGGACACTTATTCTTTGGCG<br>CTGGAAATTCAGAGAATTACCTCATTCAATTCTCGGGCAAGCGCGAGGTTAGGGCGGTTGGCTCAATCAG<br>AAATCCATTTTTAGGTTTTCAACTCCAAGCCAGTTTTGCCAAAATCCGAAGTCTGATTATCAAAACTATGA<br>TTCATTTCGGTTTTCTACGTTTTGGTTTTTGTGTGTAAATCAAAGTCGAGTTAATCTTGGTTTTGGTCAAAC<br>GTAACCCATTGAAGCTTAAGCATAACAAGGCGTTTAAGCGGGATTCATGCCGCGTGGCATTTTGGGTTTG<br>CGGTGAGTTTTGGTGGTGAAAGTGGTCTGCAGAAGCTTGGTTTATGCGGCATTCACCCCTTAACGCGGCG<br>TTATGTAGGAGCAAGCCATGAGTAAAGG
V5	*V. vulnificus* FDAARGOS_663	CTCACCATGGCTTGCTCCTTTTATGTTTAGTCACGTAAAATCAGTTGGTTGCATCTTTTCTTTGTTCCTTCG<br>TATTTCTAATTTTGGTCTTGTCGGCAAGTTAAATTCAGTGGTCGCTGTTTTCTGGACTCCAATTCGTGGGC<br>GCTAAAAATTCAGAGTGTTGCCTCATTTAAGTTCAGTGCGTTTGTGAGTCGAATGAAGCGATGTTAGTTTC<br>AACGTTTGAGCACCAGTTTTCGGTTTCTCACGCTAAAACTTCAATATCAGTACAATCAAAATTATGAGTTA<br>ATTCAGCTTTTTAGCTTCTAACTTTGGTTTTCAAAGTTAAGTCGAGTTAAGCTCTTGGTATCGTAAAACTT<br>AACCCTTTGAGCCTTAAACATAACAAGGCGTTTAAGCGGGATTCATGCCGCGTGGCATTTTTGGTTTGCG<br>TTGAGTTTTGGTGGTGAAAGAGGTCTGCGGAAACTTGGTTTAGGCGGCACTCACCCCTTAACGCAGCGTT<br>ATGTAGGAGCAAGCCATGAGTAAAGG
V6	*V. vulnificus* FDAARGOS_663	CTCACCATGGCTTGCTCCTTTTATGTTTAGTCACGTAAAAACAGTGGGTTGCATCTATTCTTTGTTTCTTCG<br>TATTTCTAATTTTGGTCTTGTCGGCAAGTTACGTTTAGTGGTCGCTGTTTTCTGGACTCCAATTCGTGGGCG<br>CTAAAAATTCAGAGAGTTGCCTCATTGAATTCCAGAATGTAGGCGAGGTGAGTGAAGTGATTTTGATTTC<br>AAAGTTAGAGCCCCATCTTTTCGATTTCTCACGTTATAAGTCAAAGTCGGCACAATCAAAATTATGATTTA<br>ATTCAGCTTTTTAGCGTCTGGTTTTGGTTTGCAAAGGTAAGTCGAGTTAAGCTATTGGTATCGCAAACTTA<br>ACCCTTTGAGCCTTAAACATAACAAGGCGTTTAAGAGGGATTCATGCCGCGTGGCATTTTTGGTATGCGG<br>TGAGTTTTGGTGGTGAAGGTGGTCTGCGGAAGCTTGGTTTATGCGGCATTCACCCCTTAACGCGGCGTTA<br>TGTAGGAGCAAGCCATGAGTAAAGG

V7	*V. vulnificus* FDAARGOS_663	CTCACCATGGCTTGCTCCTTTTATGCTTAATCACGTAAAATCAGTGGTTTATGGCTTTTCTTTGTTCCCTCA<br>GCTTTTTGACTTTGAGCTCGTCGGCAAGTTGGTTTCAGTGGGCGCTGTTTTCTGGACACTTATTCTTTGGC<br>GCTGGAAGTTCAGAGAATTGCCTAAATCAGTTTTCGCGTAAGCGCGAGGTTAGGGCAGTTGGCTTAATCA<br>GATATTTGAACTTGGCTTTTTGAGTTTTAGTCGCCAAATTTCAAAGTCCGATTTTTAAAACAATGAGTCAT<br>TTCGGTTTTCTACGTTTTGTCTTTTGTTTGTAAATCAAAGTCGAGTTAATCTTGGTTCTAGCAAAACGTAAC<br>CCATTGAAGCTTAAGCATAACAAGGCGTTCAAGAGGGATTCATGCCGCGTGGCATTTTTGGTATGCGGCG<br>AGTTTTGGTGGTGAAAGTGGTCTGCTGAAGGTTGGTTTGAGCGGCACTCACCCCTTAACGCAGCGTTATG<br>TAGGAGCAAGCCATGAGTAAAGG
V8	*V. vulnificus* FDAARGOS_663	CTCACCATGGCTTGCTCCTTTTATGCTTAATCACCTAAAATCAGTGGTTTATGGTTTTTCTTTGTTCCCTAG<br>GCTTTTCAGTTTTGTGTTTGTCGGCAAGCTGGCTTTTTTGAGCGCTGTTTTTCGGACACTTATTCTTTGGCG<br>CTAGAAATTCAGAGAATTGCCTCATTCAATTCTTGGGCAAGCGCGAGGTTAGGGCTGTTGGCTCAATCAG<br>AGATTTGATCTTAGGTTTTTTCGTTTTAGCTGCCAAATTTCAAAGTCTGATTTTCAAAATCATGAGTCATTT<br>CAGTTTTTTGGTTTTTGGCTTTTGTTTGTGAATCAAAGTCGAGTTAATCTTGTTTTTAGTAAAACGTAAGCC<br>ATTGAAGCTTCAGCATAACAAGGCGTTTAAGCGGGATTCATGCCGCGTGGCATTTTTGGTTTGCAGTGAG<br>TTTTGGTGGTGAAAGTGGTGTGCGGAAGGTTGGTTTATGCGGCATTCACCCCTTAACGCAGCGTTATGTA<br>GGAGCAAGCCATGAGTAAAGG
V9	*V. vulnificus* FDAARGOS_663	CTCACCATGGCTTGCTCCTTTTATGTTTAGTCACGTAAAATCAGTTGGTTGCATCTTTTCTTTGCTTTCTCA<br>GCTTTCTGATTTTGGTCTTGTCGGCAAGTTACGTTTAGTGGTCGCTGTTTTCTGGACTCCAATTCGTGGAC<br>GCTAAAAATTCAAAGAGTTGCCTCATTGAAATTCAGTGCGTTTGTGAGTCGAATGAAGCGATGTTAGTTT<br>CAACGTTTGAGCACCAGTTTTCGGTTTCTCTCGCTAAAATCTCAATATCAGTACAATCAAATTTATGAGTT<br>AATTCAGCTTTTTAGCTTCTAACTTTGGTTTTCAAAGATAAGTCGAGTTAAGCTCTTGGTATTGCAAAACT<br>TACCCCTTTGAGTCTTAAACATAACAAGGCGTTTAAGAGGGATTCATGCCGCGTGGCATTTTTGGTTTGCA<br>GTGAGTTTTGGTGGTGAAAGTGCTCTGCGGAAAGTTGATTTATGCGGCATTCACCCCTTAACGCAGCGTT<br>ATGTAGGAGCAAGCCATGAGTAAAGG
V10	*V. vulnificus* FDAARGOS_663	CTCACCATGGCTTGCTCCTTTTATGCTTAATCACGTAAAATCAGTGGTTTATGGTTTTTCTTTGTTCCCTCG<br>GCTTTTCATTTTTGTGTTTGTCGGCAAGTTGGCTTTTGTGAGCGCTGTTTTTCGGACACTTATTCTTTGGCG<br>CTGGAAATTCAGAGAATTGCCTCATTCAATTCTTGGGTAAGCGCGAGGTTAGGGCGGTTGGCTCAATCAG<br>AAATCCATTTCTAGGTTTTCAAACTTCAAAATGGATTTGCCAAAATCCCGAACCTGAATATCAAAACTAT<br>GATTCATTTCAGTTTTCTACGTTTTGGTTTTTGTTTGTAAATCAAAGTCGAGTTAATCTTGTTTTTAGTAAA<br>ACGTAAGCCATTGAAGCTTAAGCATAACAAGGCGTTTAAGAGGGATTCATGCCGCGTGGCATTTTTGGTT<br>TGCAGTGAGTTTTGGTGGTGAAAATGGTCTGCGGAAAGTTGGTTTATGCGGCATTCACCCCTTAACGCAG<br>CGTTATGTAGGAGCAAGCCATGAGTAAAGG

V11	*V. vulnificus* FDAARGOS_663	CTCACCATGGCTTGCTCCTTTTATGTTTAATCACGTAAAATCAGCCGGTTGCATCTTTTCTTTGTTCCTTCG CATTTCCAATTTTGGTCTTGTCGGCAAGTTGGCTTTCGGTGGTCGCTGGTTTCTGGACTCCAATTCGTGGG CGCGAAAAATTCTGAGAGTTGCCTCATTAAAGCTCAGTGCGTTTGTGAGTCGAATAAAGCGTCTTAGTTT CAAAGTTTGAGCTCCAGCTTTCGGTTTCTCACGCTAAAAATCAAAATCATTAGAATCAAATTTATGAGTT AATTCAGCTTTTTAGCGTCTGATTTTGGTTTTTAAAGGTAACTCGAGTTAAGCTCTTGGTATCGCAAAACT TAACCCTTTGAGCCTTAAACATAACAAGGCGTTTAAGAGGGATTCATGCCGCGTGGCATTTTTAGTATGC GGTGAGTTTTGGTGGTGAAAGTGGTCTGCGGAAACTTGGTTTAGGCGGCACTCACCCCTTAACGCGGCGT TATGTAGGAGCAAGCCATGAGTAAAGG
V12	*V. vulnificus* FDAARGOS_663	CTCACCATGGCTTGCTCCTTTTATGCTTAATCACGTAAAATCAGTTGGTTATGGTTTTTCTTTGTTCCCTCG GCTTTTCAGTTCGGTGTTTGTCGGCAAGTTGGCTTCTTAGAGCGCTGTTTTTCGGACATTTATTCTTTGGCG CTGGAAATTCAGAGAGTTGCCTCAGTCAATTTTCGGGTAAGCGCTAGGTTAGGGTAGTTGGCTCAATCAG AAATTTGATCTTAGGTTTTTTCGTTTTGGCTGCCACAGTTCAAAGTCTGATTTTCAAAATCATGAGTCATTT CAGTTTTTTGGTTTTTGGCTTTTGTGTGTAAATCAAAGTCGAGTTAATCTTGGTTTTGGTCAAACGTAACC CATTGAAGCTTAAGCATAACAAGGCGTTTAAGAGGGATTCATGCCGCGTGGCATTTTTGGTTTGCAGTGA GTTTTGGTGGTGAAAATGGTCTGCGGAAAGTTGGTTTATGCGGCATTCACCCCTTAACGCAGCGTTATGT AGGAGCAAGCCATGAGTAAAGG
V13	*V. vulnificus* FDAARGOS_663	CTCACCATGGCTTGCTCCTTTTATGTTTAGTCACGTAAAATCAGTTGGTTGCAGATTTTTTTGCTTTCTAAT TTTGGCCTTGTCGGCAAGCTGGCTTTCGGTGGTCGCTGTTTTCTGGACTCCAATTCGTGGGCGCTAAAAAT TCTGAGAGTTGCCTCATTAAAGCTCAGTGCGTTTGTGAGTCGAATAAAGCGTCTTAGTTTCAAAGTTTGA GCTCTAGTTTTCTGTTTCTCACGTTAAAACTCAAAGTTAGCACAATCAAATTTATGATTTAATTCAGCTTTT TAGCGTCTGGTTTTGGTTTGCAAAGGTAAGTCGAGTTAAGCTATTGGTATCGCAAACTTAACCCTTTGAGC CTTAAACATAACAAGGCGTTTAAGCGGGATTCATGCCGCGTGGCATTTTTTGTTTGCGGTGATTTTTGGTG GTGAAAGTGGTCTGCAGAAGCTTGGTTTATGCGGCATTCACCCCTTAACGCGGCGTTATGTAGGAGCAAG CCATGAGTAAAGG
*lacZ*450	*V. cholerae* N16961	AAACCCGCACATCGTTAAATGGCACTGCCGTACACCCCATGTTCCTTTGCACAGTTATCGCACTGAGCAG GAGGCTCGTTTGGATGTTGGGGGGAATCGCCAATCTCTAAATGGTCAGTGGCGGTTTGCTCTGTTTGAGA AGCCAGAAGCGGTTGAGCCTGCGGTGATAGACCCGGATTTCGATGATAGCGCTTGGGCGCACATTCCTGT ACCGAGTAACTGGCAGATGCAAGGCTTTGATAAGCCGATTTACACCAATATCCAATATCCATTTGCGGAT CGGCCGCCTTACGTGCCGCAAGATAATCCAACCGGCTGTTATCGCCACCGTTTTACACTGGAAAAACAAG CGCTAACCGAGTCCATTCGCATTGTATTTGATGGGGTCAATTCGGCATTTCATCTGTGGTGCAATGGTCAT TGGGTCGGTTATTCGCAAGATAGCCGCTT

*lacZ*550	*V. cholerae* N16961	AAACCCGCACATCGTTAAATGGCACTGCCGTACACCCCATGTTCCTTTGCACAGTTATCGCACTGAGCAG<br>GAGGCTCGTTTGGATGTTGGGGGGAATCGCCAATCTCTAAATGGTCAGTGGCGGTTTGCTCTGTTTGAGA<br>AGCCAGAAGCGGTTGAGCCTGCGGTGATAGACCCGGATTTCGATGATAGCGCTTGGGCGCACATTCCTGT<br>ACCGAGTAACTGGCAGATGCAAGGCTTTGATAAGCCGATTTACACCAATATCCAATATCCATTTGCGGAT<br>CGGCCGCCTTACGTGCCGCAAGATAATCCAACCGGCTGTTATCGCCACCGTTTTACACTGGAAAAACAAG<br>CGCTAACCGAGTCCATTCGCATTGTATTTGATGGGGTCAATTCGGCATTTCATCTGTGGTGCAATGGTCAT<br>TGGGTCGGTTATTCGCAAGATAGCCGCTTGCCTGCCGAGTTTGAGTTAACCCCTTATCTACAAGAGGGTG<br>AAAACCTGTTGGTGGCCATGGTGCTGCGCTGGTCTGATGGCTCTTATTTGGAAGACCAA
*lacZ*650	*V. cholerae* N16961	AAACCCGCACATCGTTAAATGGCACTGCCGTACACCCCATGTTCCTTTGCACAGTTATCGCACTGAGCAG<br>GAGGCTCGTTTGGATGTTGGGGGGAATCGCCAATCTCTAAATGGTCAGTGGCGGTTTGCTCTGTTTGAGA<br>AGCCAGAAGCGGTTGAGCCTGCGGTGATAGACCCGGATTTCGATGATAGCGCTTGGGCGCACATTCCTGT<br>ACCGAGTAACTGGCAGATGCAAGGCTTTGATAAGCCGATTTACACCAATATCCAATATCCATTTGCGGAT<br>CGGCCGCCTTACGTGCCGCAAGATAATCCAACCGGCTGTTATCGCCACCGTTTTACACTGGAAAAACAAG<br>CGCTAACCGAGTCCATTCGCATTGTATTTGATGGGGTCAATTCGGCATTTCATCTGTGGTGCAATGGTCAT<br>TGGGTCGGTTATTCGCAAGATAGCCGCTTGCCTGCCGAGTTTGAGTTAACCCCTTATCTACAAGAGGGTG<br>AAAACCTGTTGGTGGCCATGGTGCTGCGCTGGTCTGATGGCTCTTATTTGGAAGACCAAGATATGTGGTG<br>GCTGAGTGGCATCTTTCGCGATGTGTATCTCTACCGCAAGCCGATACTCGCGATTGAAGATTTTTTATCC<br>GCACTGAATTAGATGCGC

Supplementary Table S2. Primers used in this study.

Oligo	Sequence (5' → 3')	Description
bb_pSU38_long_F	CTCACCATGGCTTGCTCCTTAGG AGCAAGCCATGAGTAAAGG	Construction of pDProm. Amplification of pA369 backbone.
bb_pSU38_long_R	GGACGAGCTGTACAAGTAAAAT TGCGTTGCGCTCACTGC	Construction of pDProm. Amplification of pA369 backbone.
bb_pSU38_F	AGGAGCAAGCCATGAGTAAAGG	Amplification of pDProm backbone for cassettes cloning.
bb_pSU38_mCherry _R	AAGGAGCAAGCCATGGTGAG	Amplification of pDProm backbone for cassettes cloning.
pDProm_lacZ_F	CTCACCATGGCTTGCTCCTTAAA CCCGCACATCGTTAAAT	Amplification of *lacZ* gene.
pDProm_lacZ450_R	CCTTTACTCATGGCTTGCTCCTA AGCGGCTATCTTGCGAATAA	Amplification of *lacZ* gene. Construction of pDProm-*lacZ*450.
pDProm_lacZ550_R	CCTTTACTCATGGCTTGCTCCTTT GGTCTTCCAAATAAGAGCCA	Amplification of *lacZ* gene. Construction of pDProm-*lacZ*550.
pDProm_lacZ650_R	CCTTTACTCATGGCTTGCTCCTG CGCATCTAATTCAGTGCG	Amplification of *lacZ* gene. Construction of pDProm-*lacZ*450.
MRVII	GGTTTCCCGACTGGAAAGCG	Check of insert presence in pDProm.
MFD	CGCCAGGGTTTTCCCAGTCAC	Check of insert presence in pDProm.
bb_pSU38_dfrB9_F	CGTTAGGCGTCGAGCTGCTCTAG ACAGCGCCGTCGTTGC	Construction of pDProm-*drfB9*. Backbone amplification.
bb_pSU38_dfrB9_R	TTCGCATTGCGGGGCCTAACAAG GAGCAAGCCATGGTGAG	Construction of pDProm-*drfB9*. Backbone amplification.
dfrB9_pSU38_F	CTCACCATGGCTTGCTCCTTGTT AGGCCCCGCAATGCGAA	Construction of pDProm-*drfB9*. Cassette amplification.
dfrB9_pSU38_R	GCAACGACGGCGCTGTCTAGAG CAGCGTCGACGCCTAACG	Construction of pDProm-*drfB9*. Cassette amplification.
Plac_dfrB9_F	TCACACAGGAAACAGCTATGGT TAGGCCCCGCAATGCGAA	Construction of pDProm P_{lac}-*drfB9*. Cassette amplification.
bb_pSU38_plac_R	TTCGCATTGCGGGGCCTAACCAT AGCTGTTTCCTGTGTGA	Construction of pDProm P_{lac}-*drfB9*. Backbone amplification.
bb_dfrB9_E7_F	CACTTAACGCGGCGTTATGTGTT AGGCCCCGCAATGCGAA	Construction of pDProm E7-*drfB9*. Backbone amplification.

E7_dfrB9_R	TTCGCATTGCGGGGCCTAACACA TAACGCCGCGTTAAGTG	Construction of pDProm E7-*drfB9*. Cassette amplification.
bb_dfrB9_D11_F	GTGTTATGCAGGCGTTATGTGTT AGGCCCCGCAATGCGAA	Construction of pDProm D11-*drfB9*. Backbone amplification.
D11_dfrB9_R	TTCGCATTGCGGGGCCTAACACA TAACGCCTGCATAACAC	Construction of pDProm D11-*drfB9*. Cassette amplification.
bb_dfrB9_C12_F	CCCTTAGGCGGGCGTTATGTGTT AGGCCCCGCAATGCGAA	Construction of pDProm C12-*drfB9*. Backbone amplification.
C12_dfrB9_R	TTCGCATTGCGGGGCCTAACACA TAACGCCCGCCTAAGGG	Construction of pDProm C12-*drfB9*. Cassette amplification.
bb_dfrB9_D1_F	CCCTTAGGCGGGCGTTATGTGTT AGGCCCCGCAATGCGAA	Construction of pDProm D1-*drfB9*. Backbone amplification.
D1_dfrB9_R	TTCGCATTGCGGGGCCTAACACA TAACGCCCGCCTAAGGG	Construction of pDProm D1-*drfB9*. Cassette amplification.
bb_dfrB9_F11_F	CCCTTAACGCGGCGTTATGTGTT AGGCCCCGCAATGCGAA	Construction of pDProm F11-*drfB9*. Backbone amplification.
F11_dfrB9_R	TTCGCATTGCGGGGCCTAACACA TAACGCCGCGTTAAGGG	Construction of pDProm F11-*drfB9*. Cassette amplification.
C7_dfrB9_F	GCCTCACCTCGAACGTTATGCTT AATCCATAAAAATCAGC	Construction of pDProm-C8-dfrB9-C7. Cassette amplification.
C7_dfrB9_R	GAGCAGCGTCGACGCCTAACGC CCGCCTAAGGG	Construction of pDProm-C8-dfrB9-C7. Cassette amplification.
F1_dfrB9_F	TTTGCGCCTCACCTCGAACGTTA TACGAAATCAATGACGTCC	Construction of pDProm-C8-dfrB9-F1. Cassette amplification.
F1_dfrB9_R	AGAGCAGCGTCGACGCCTAACA CCTGCATAACACGTTTGC	Construction of pDProm-C8-dfrB9-F1. Cassette amplification.
pSU_dfrB9_F	TTAGGCGTCGACGCTGC	Construction of pDProm-C8-dfrB9-F1 and pDProm-C8-dfrB9-C7. Backbone amplification.
pSU_dfrB9_R	CGTTCGAGGTGAGGCG	Construction of pDProm-C8-dfrB9-F1 and pDProm-C8-dfrB9-C7. Backbone amplification.
gyrA_rt_F	GAGCCAAAGTTACCTTGGCC	*gyrA* amplification for RT-qPCR

gyrA_rt_R	AATGTGCTGGGCAACGACTG	*gyrA* amplification for RT-qPCR
gyrA_PRB	[5SUN]-CACCCTCAT-[ZEN]-GGTGACAGTGCGGTTT-[IBFQ]	*gyrA* probe for RT-qPCR
gfp_rt_F	GGTGAAGGTGATGCAACATA	*gfp* amplification for RT-qPCR
gfp_rt_R	GAAGACCATACGCGAAAGTAG	*gfp* amplification for RT-qPCR
gfp_PRB	[6FAM]-ACCTGTTCC-[ZEN]-ATGGCCAACACTTGTCA-[IBFQ]	*gfp* probe for RT-qPCR
mCherry_rt_F	CTACTTGAAGCTGTCCTTCC	*mCherry* amplification for RT-qPCR
mCherry_rt_R	TAGATGAACTCGCCGTCT	*mCherry* amplification for RT-qPCR
mCherry_PRB	[56FAM]-TCAAGTGGG-[ZEN]-AGCGCGTGATGAACT-[IBFQ]	*mCherry* probe for RT-qPCR
F1_gsp1_R	GCGGCAAACCCAAAGAAATCTA	5'RACE of cassette F1
F1_gsp2_R	GAATGCCGAATGTAGCAAATCG	5'RACE of cassette F1
F1_gsp3_F	CAGTTAGTAAGGCAGTGTCAATGT	5'RACE of cassette F1
F1_gsp4_R	CATCTTATCACAGCTAAAACAACAC	5'RACE of cassette F1
F1_ant_gsp1_R	CTCCTTTTATACGAAATCAATGACG	5'RACE of cassette F1 (antisense)
F1_ant_gsp2_R	CAGTTAGTAAGGCAGTGTCAATGT	5'RACE of cassette F1 (antisense)
F1_ant_gsp1_R	TTAACGCGGCAAACCCAAAG	5'RACE of cassette F1 (antisense)
F2_gsp1_R	CCACAATAACACGTGCGAAAGA	5'RACE of cassette F2
F2_gsp2_R	GACTGACTTACAAAACCAATTATACTC	5'RACE of cassette F2
F2_gsp3_F	GTGTTCGTTTCATCAGGTTCGG	5'RACE of cassette F2
F2_gsp4_R	TTATACGAGCCTTGCTTTCGC	5'RACE of cassette F2
C5_gsp1_R	GGCTGGCAACGCATTACTACT	5'RACE of cassette C5
C5_gsp2_R	AAACCCAAACACAACAACCGT	5'RACE of cassette C5
C5_gsp3_F	TGATGCTGATTTGGTCGAACG	5'RACE of cassette C5
C5_gsp4_R	TCGTAGCCTATTGTTTAACAATGGC	5'RACE of cassette C5
V11_gsp1_R	GGTGAGTGCCGCCTAAAC	5'RACE of cassette V11
V11_gsp2_R	CAAGTTTCCGCAGACCACTTTC	5'RACE of cassette V11
V11_gsp3_F	ATCACGTAAAATCAGCCGGTTG	5'RACE of cassette V11
V11_gsp4_R	CCAAGAGCTTAACTCGAGTTACC	5'RACE of cassette V11
V7_gsp1_R	CTCAAACCAACCTTCAGCAGAC	5'RACE of cassette V7
V7_gsp2_R	CACTTTCACCACCAAAACTCGC	5'RACE of cassette V7
V7_gsp3_F	GGTTTATGGCTTTTCTTTGTTCCC	5'RACE of cassette V7

V7_gsp4_R	TGCTAGAACCAAGATTAACTCG AC	5'RACE of cassette V7
M13_F	CTGGCCGTCGTTTTAC	Check insert in pTOPO-TA
M13_R	GTCATAGCTGTTTCCTG	Check insert in pTOPO-TA

Supplementary Table S3. Putative promoter sequences detected with 5'RACE

Cassette	TSS position	-10 box	-35 box
F1	35	TTATAA	TTATAC
	122 (as)	ACATGA	CTCATT
	191	GTGCCG	TTTAGC
	194	CCGAGG	AGCCAA
	197	AGGGGG	CAATGA
	240 (as)	AGCTCC	CTACAG
	283 (as)	TGAACG	AGGGCT
	317	GTTCGA	TGTAGA
	364	TGTTTT	TTCATA
	375	TGATAA	CATATC
F2	239	TTTTCC	TTAGCC
	408	TAATTA	TTGAGC
	430	TGGTAA	TTACAG
	440	GTTCTT	TTTACA
	455	TAATTG	TGGTAA
	466	AAGCAA	TTACCA
	474	GTATAA	AAGTTA
	490	ATCAAC	GAAAGC
	510	ATTCGG	AAGCAA
C5	220	AAAATG	TTCCGC
	293	CGTTTT	TAACCG
	337	CGTGTG	CAACGT
	346	GCTTGA	TTGGAC
	367	TCAAAC	TTGATT
V7	12	TTATGC	-
	107	GGGCGC	AGCTCG
	134	TTGGCG	GCGCTG
	159	TAAATC	TTCAGA
	165	TCAGTT	TGGAAG
	185	AGGTTA	CTAAAT
	252	AAGTCC	TGAGTT
	261	TTAAAA	TAGTCG
V13	106	GTGGTC	TGGTCT
	195	TAAAGC	TGCGTT
	222	TCCAGC	GCGTCT
	226	CTTTCG	TTAGTT
	227	TTTCGG	TTAGTT
	244	TAAAAA	TTCGGT

(as): Antisense

Supplementary Table S4. Putative promoter position and scores predicted by BPROM

<table>
<tr><th>Gene-less cassette or promoter</th><th>Strand</th><th>Score -10 box</th><th>Score -35 box</th><th>-10 box</th><th>-35 box</th><th>LDF</th></tr>
<tr><td>P_CS</td><td>Sense</td><td>63</td><td>66</td><td>TCGTAAACT</td><td>TTGACA</td><td>5,97</td></tr>
<tr><td>P_{lac}</td><td>Sense</td><td>44</td><td>47</td><td>TCGTATGTT</td><td>TTTACA</td><td>3,94</td></tr>
<tr><td>P_CW</td><td>Sense</td><td>47</td><td>29</td><td>TCGTAAGCT</td><td>TGGACA</td><td>3,10</td></tr>
<tr><td rowspan="2">C1</td><td>Sense</td><td>60</td><td>40</td><td>TTGTAAATT</td><td>TTCAAA</td><td>4,64</td></tr>
<tr><td>Antisense</td><td>32</td><td>61</td><td>GTGTATTAA</td><td>TTGCCA</td><td>2,37</td></tr>
<tr><td rowspan="3">C2</td><td>Sense</td><td>50</td><td>38</td><td>AGTTCTAAT</td><td>TTCATA</td><td>3,56</td></tr>
<tr><td rowspan="2">Antisense</td><td>67</td><td>66</td><td>TGTTATGCT</td><td>TTGACA</td><td>6,50</td></tr>
<tr><td>20</td><td>24</td><td>ATCGACAAT</td><td>CTGAAT</td><td>0,91</td></tr>
<tr><td rowspan="3">C3</td><td>Sense</td><td>34</td><td>36</td><td>AGGTTGAAT</td><td>TTGATC</td><td>2,59</td></tr>
<tr><td rowspan="2">Antisense</td><td>69</td><td>40</td><td>TTTTAAACT</td><td>TTGTTT</td><td>4,15</td></tr>
<tr><td>5</td><td>38</td><td>TTTCAGAAC</td><td>TTGAAC</td><td>0,34</td></tr>
<tr><td rowspan="4">C4</td><td rowspan="2">Sense</td><td>70</td><td>0</td><td>GCGTAAAAT</td><td>GTGCGT</td><td>3,59</td></tr>
<tr><td>32</td><td>30</td><td>CCTGAAAAT</td><td>TTATCA</td><td>1,12</td></tr>
<tr><td rowspan="2">Antisense</td><td>38</td><td>66</td><td>TGTTAGCTT</td><td>TTGACA</td><td>2,76</td></tr>
<tr><td>24</td><td>38</td><td>CAGGACAAT</td><td>TTGAAC</td><td>1,60</td></tr>
<tr><td rowspan="3">C5</td><td>Sense</td><td>70</td><td>15</td><td>GCGTAAAAT</td><td>GTGCTT</td><td>2,74</td></tr>
<tr><td rowspan="2">Antisense</td><td>34</td><td>45</td><td>TTTAACAAT</td><td>TTGTTA</td><td>3,61</td></tr>
<tr><td>77</td><td>-6</td><td>CGCTAAAAT</td><td>AAGCCA</td><td>3,44</td></tr>
<tr><td rowspan="3">C6</td><td>Sense</td><td>39</td><td>46</td><td>CGCTGAAAT</td><td>TTCACA</td><td>3,22</td></tr>
<tr><td rowspan="2">Antisense</td><td>42</td><td>66</td><td>TGTTAGTTT</td><td>TTGACA</td><td>2,37</td></tr>
<tr><td>47</td><td>34</td><td>TAGCAAAAT</td><td>TTTCTA</td><td>2,21</td></tr>
<tr><td rowspan="4">C7</td><td rowspan="2">Sense</td><td>56</td><td>30</td><td>CTGTAAATT</td><td>ATGAAA</td><td>3,82</td></tr>
<tr><td>42</td><td>39</td><td>TGGTGTTAT</td><td>TTGTTG</td><td>0,50</td></tr>
<tr><td rowspan="2">Antisense</td><td>67</td><td>66</td><td>TGTTATGCT</td><td>TTGACA</td><td>6,50</td></tr>
<tr><td>-6</td><td>55</td><td>GAACGAAAT</td><td>TTGAAT</td><td>1,04</td></tr>
<tr><td rowspan="3">C8</td><td>Sense</td><td>70</td><td>15</td><td>GCGTAAAAT</td><td>GTGCTT</td><td>2,74</td></tr>
<tr><td rowspan="2">Antisense</td><td>34</td><td>45</td><td>TTTAACAAT</td><td>TTGTTA</td><td>3,61</td></tr>
<tr><td>77</td><td>-6</td><td>CGCTAAAAT</td><td>AAGCCA</td><td>3,44</td></tr>
<tr><td rowspan="4">C9</td><td rowspan="2">Sense</td><td>70</td><td>7</td><td>GCGTAAAAT</td><td>GTGTTT</td><td>2,89</td></tr>
<tr><td>40</td><td>10</td><td>CTGAAAAAT</td><td>ATCAAA</td><td>1,04</td></tr>
<tr><td rowspan="2">Antisense</td><td>41</td><td>40</td><td>TTGAATACT</td><td>TTCAAA</td><td>2,32</td></tr>
<tr><td>38</td><td>66</td><td>TGTTAGCTT</td><td>TTGACA</td><td>2,28</td></tr>
<tr><td>C10</td><td>Sense</td><td>70</td><td>15</td><td>GCGTAAAAT</td><td>GTGCTT</td><td>4,37</td></tr>
</table>

	Antisense	38	22	GACTAAGCT	ATGATG	2,47
P1	Sense	60	25	GGGTAATCT	TTGGTT	5,02
		56	24	TGGTATTGT	TTTCTA	1,84
	Antisense	56	3	TGACATAAT	CTACCA	2,93
		45	61	AGTCAAACT	TTGCCA	2,02
P2	Sense	59	39	GTGTAACAT	TTTATA	3,77
	Antisense	38	29	AGCTAACTT	TGGACA	4,09
P3	Sense	69	55	GCTTAAAAT	TTGCAA	3,97
		54	31	TGCTATTTT	TTTCAT	1,35
	Antisense	39	55	TGTTTTGAT	TTGCAA	3,40
		21	24	AAACATGAT	TAGCCA	0,79
P4	Sense	60	39	GGGTAATCT	TTGTTG	5,54
	Antisense	37	60	CCTTATAAG	TTGAAA	4,92
		28	61	AGGTAAGAA	TTGCCA	1,05
V1	Sense	57	40	AGTTAATCT	TTGTTT	4,55
	Antisense	67	25	TGTTATGCT	ATGAAT	2,63
		13	55	TGAAAAGCT	TTGCCG	1,01
V2	Sense	22	60	GAGTCGAAT	TTGAAA	2,36
	Antisense	50	20	AGTTTTAAT	CTGAAT	2,63
V3	Sense	60	33	CAGTACAAT	TTCTCA	3,21
	Antisense	57	25	TGTTATGTT	ATGAAT	3,06
V4	Sense	57	25	AGTTAATCT	TTGTGT	3,87
	Antisense	67	25	TGTTATGCT	ATGAAT	2,51
V5	Sense	60	33	CAGTACAAT	TTCTCA	3,14
	Antisense	57	25	TGTTATGTT	ATGAAT	3,06
V6	Sense	58	7	AATTATGAT	GTCAAA	4,06
	Antisense	57	25	TGTTATGTT	ATGAAT	3,06
V7	Sense	57	40	AGTTAATCT	TTGTTT	4,07
	Antisense	67	25	TGTTATGCT	ATGAAT	2,95
V8	Sense	57	40	AGTTAATCT	TTGTTT	4,45
	Antisense	46	38	ACCTAAGAT	TTGGCA	3,03
V9	Sense	77	21	CGCTAAAAT	TTGAGC	4,20
	Antisense	57	25	TGTTATGTT	ATGAAT	2,96
		7	55	CAGAAAGCT	TTGCCG	0,59
V10	Sense	57	40	AGTTAATCT	TTGTTT	4,66
	Antisense	67	25	TGTTATGCT	ATGAAT	2,70
V11	Sense	58	33	CATTAGAAT	TTCTCA	2,64

	Antisense	57	25	TGTTATGTT	ATGAAT	3,06
		18	32	AGAAAAGAT	TTGGAA	0,58
V12	Sense	57	25	AGTTAATCT	TTGTGT	3,99
	Antisense	67	25	TGTTATGCT	ATGAAT	2,57
V13	Sense	65	5	ATTTATGAT	CTCAAA	3,35
	Antisense	57	25	TGTTATGTT	ATGAAT	3,71
		21	55	CAAAAAAAT	TTGCCG	0,59
F1	Sense	61	40	ATTTATATT	TTGTTT	9,42
		46	36	TTTTACAGT	TTTAAT	2,63
	Antisense	53	55	ACTTAATAT	TTGAAT	6,49
		76	66	CTGTAAAAT	TTGACA	6,20
F2	Sense	77	47	GAGTATAAT	TTTACA	6,16
		55	31	AACTATTAT	TTTCAT	3,95
	Antisense	44	36	GAGCAAAAT	TTTAAT	4,65
		40	46	TGGTTCAAT	TTCACA	2,81
F3	Sense	76	27	CAGTATAAT	TTCTCG	4,70
		31	11	TTTTAAACG	TTCCTC	2,57
	Antisense	47	52	CGGCAAACT	TTGATG	4,71
		70	17	GAGTAAAAT	ATGCTG	2,41

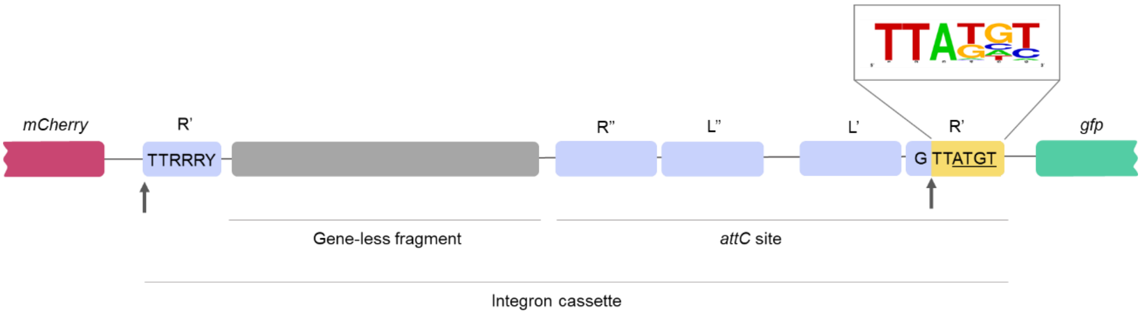

Supplementary Figure S1. General structure of the synthesized gene-less cassettes cloned in pDProm . The gene-less DNA fragment for each *Vibrio* spp. was selected including the conserved recombination site R' (G↓TTRRRY) from the recombination point marked with an arrow (TTRRRY), the gene-less fragment, and the *attC* site containing the binding domains R'', L'', L' and R'. After the GTT of the R' recombination point (vertical arrow), the nucleotides ATGT were added to each fragment (yellow box). ATGT were the most prevalent nucleotides from an aligment of all *attC* sites selected in this study (inset).

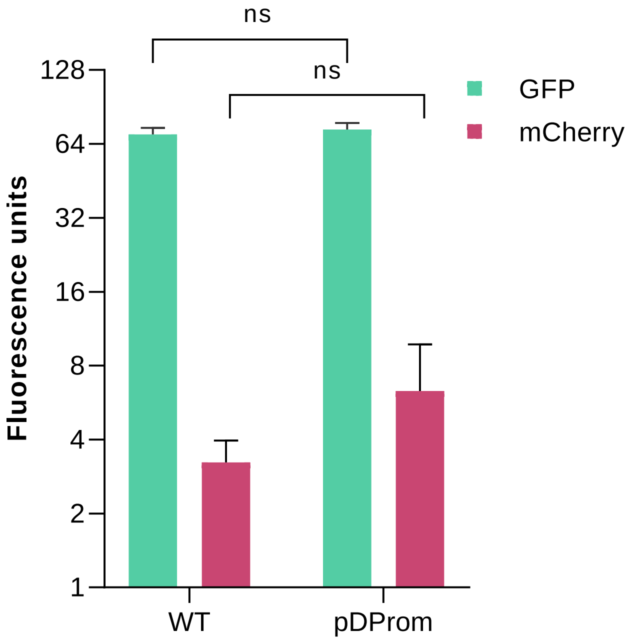

Supplementary Figure S2. Comparison of fluorescence raw values between *V. cholerae* WT and *V. cholerae* carrying the empty pDProm plasmid. The fluorescence raw values of both *gfp* (sense) and *mCherry* (antisense) were measured using flow cytometry in *V. cholerae* WT and *V. cholerae* containing the plasmid pDProm without any insert. Error bars represent standard deviation of fluorescence measurements of three biological replicates with two technical replicates each. Paired t-test was performed by comparing the measures obtained for both strains; ns: not significant.

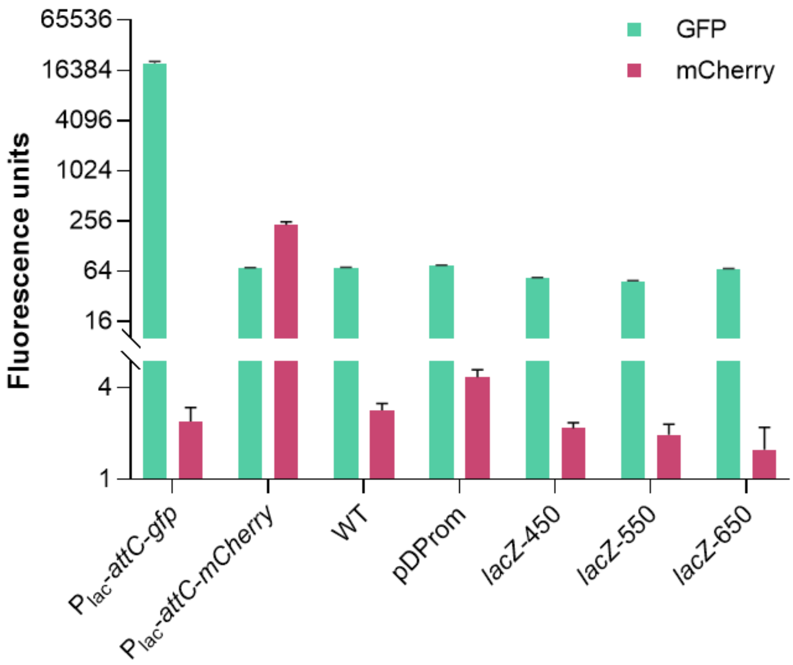

Supplementary Figure S3. Fluorescence raw values of *V. cholerae* with pDProm and control DNA fragments. The fluorescence raw values of both *gfp* (sense) and *mCherry* (antisense) were measured using flow cytometry in *V. cholerae* N16961 strains carrying pDProm with control DNA fragments of different sizes (450-, 550-, and 650-bp) originated from the *lacZ* gene. Fluorescence levels were also measured in *V. cholerae* carrying pDProm-P_{lac}-*attC-gfp*, pDProm-P_{lac}-*attC-mCherry*, the wildtype strain (WT), and the one harboring the empty pDProm. Error bars represent standard deviation of fluorescence measurements of three biological replicates with two technical replicates each.

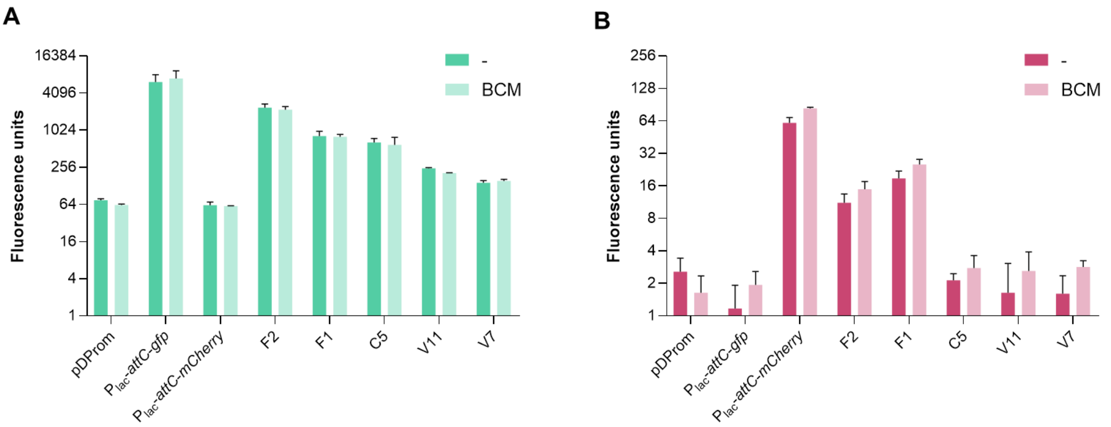

Supplementary Figure S4. Effect of bicyclomycin (BCM) on fluorescence raw levels of gene-less cassettes. The fluorescence raw values of both (**A**) *gfp* (sense) and (**B**) *mCherry* (antisense) were measured using flow cytometry in *V. cholerae* strains carrying pDProm and derivatives with or without 2h-incubation with bicyclomycin (50 µg/mL). Error bars represent standard deviation of fluorescence measurements of three technical replicates.

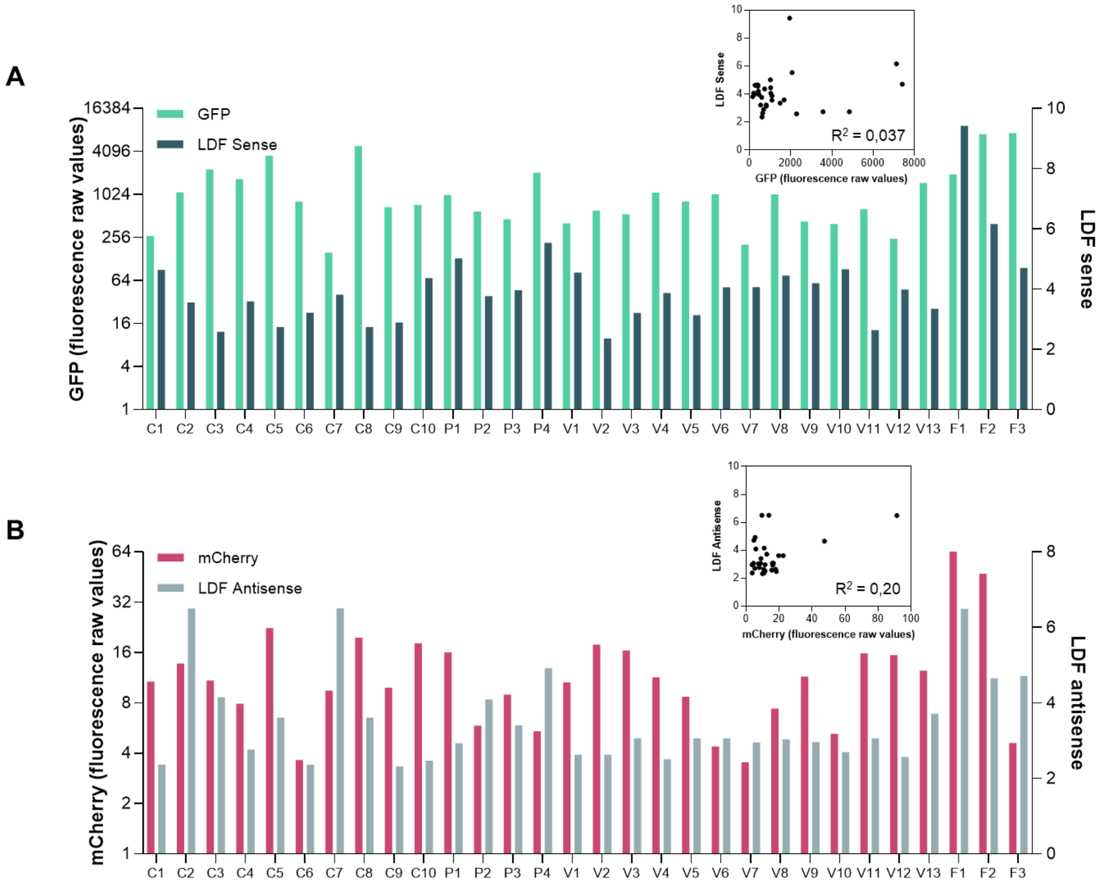

Supplementary Figure S5. Predicted promoter strength for the *Vibrionaceae* gene-less cassettes compared with the observed expression. Mean of raw *gfp* fluorescence values (left Y axis) and predicted LDF score of the sense strand by BPROM (right Y axis) of all the selected *Vibrionaceae* cassettes (**A**). Mean of raw *mCherry* fluorescence values (left Y axis) and predicted LDF score of the antisense strand by BPROM (right Y axis) of all the selected *Vibrionaceae* cassettes (**B**). GFP and mCherry fluorescence values do not correlate with the sense and antisense LDF values, respectively (insets: R^2=0,037; R^2=0,20). The fluorescence mean is calculated from measurements of three biological replicates with two technical replicates each. LDF, linear discriminant function.

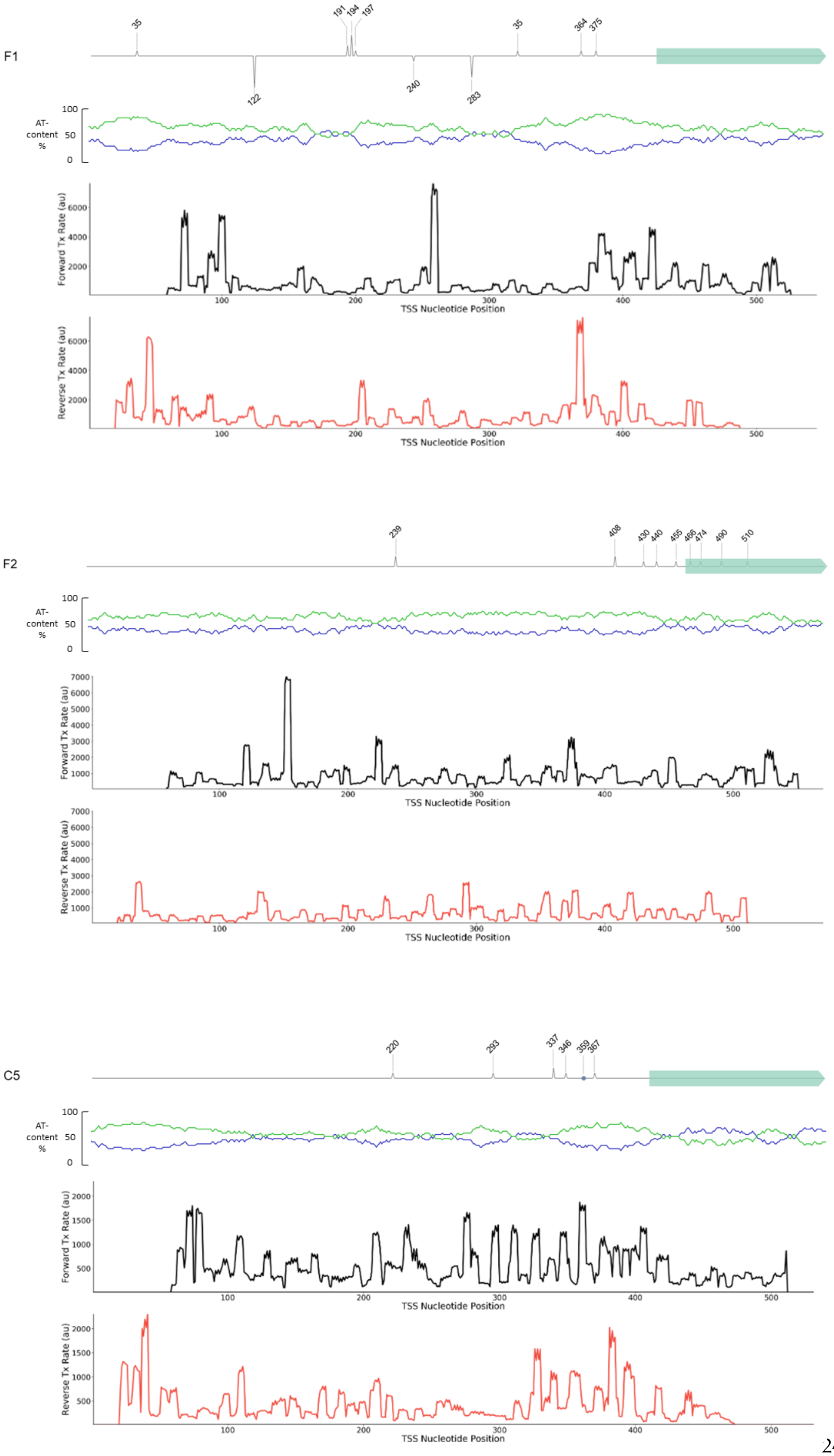
F1
35
191
194
197
35
364
375
122
240
283
AT-content %
100
50
0
Forward Tx Rate (au)
Reverse Tx Rate (au)
TSS Nucleotide Position
F2
239
408
430
440
455
466
474
490
510
C5
220
293
337
346
359
367

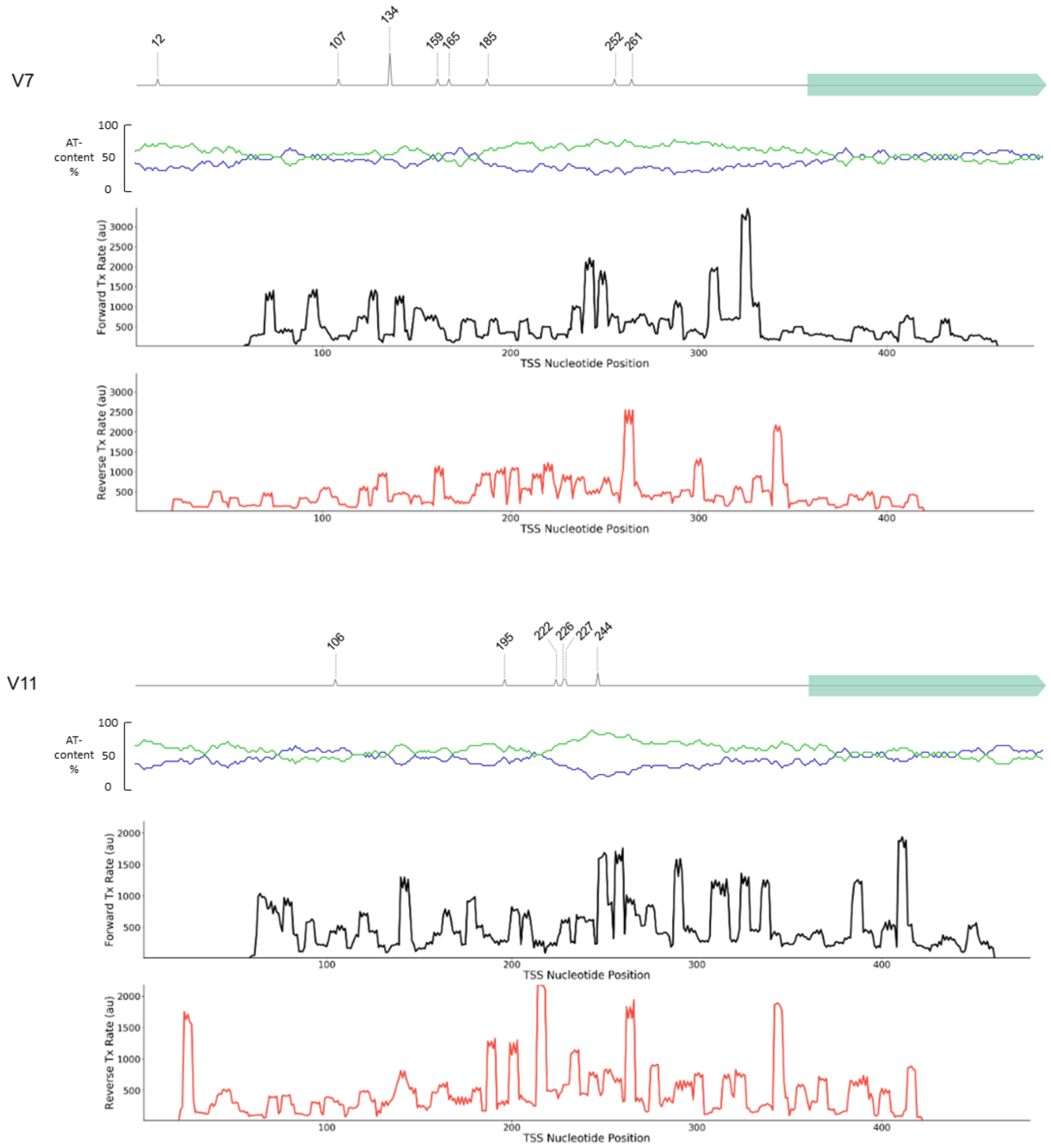

Supplementary Figure S6. Comparison of detected transcription start sites *in vitro* and *in silico* in gene-less cassettes. The transcription start sites (TSSs) identified through 5' RACE along the selected cassettes are overlaid with the *in silico* predictions by De Novo DNA software, presented as peaks indicating transcription rates. The experimentally detected TSSs through 5' RACE do not match with those identified by the De Novo DNA software. Graphs illustrating the percentage of AT-content (green curve) and GC-content (blue curve) are also provided for each cassette.

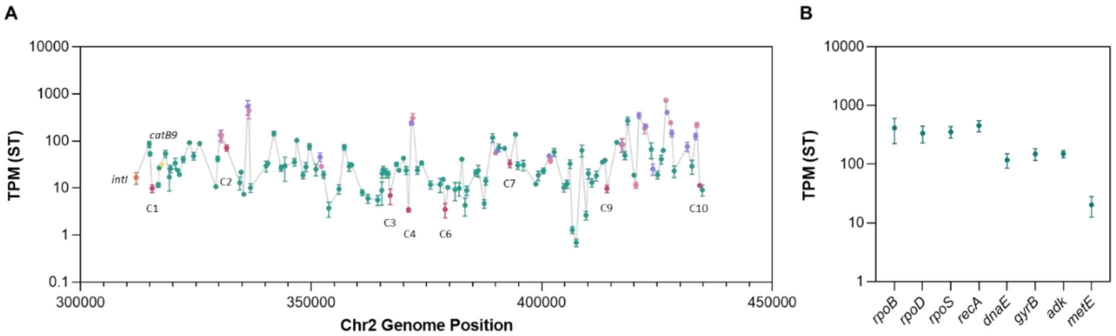

Supplementary Figure S7. Expression levels of *V. cholerae* N16961 superintegron gene cassettes from BioProject PRJNA420494. Expression levels represented as transcripts per kilobase million (TPM) of gene cassettes along the superintegron array were calculated from RNA-seq data available in databases in stationary (ST) growth phase (**A**). TPM values of eight housekeeping genes were used as a reference (**B**). TPM values of genes-less cassettes (magenta), toxin-antitoxin systems (mauve and pink), integrase (orange), and the chloramphenicol resistance gene *catB9* (yellow), are highlighted. Error bars represent the standard deviation of three independent biological replicates.

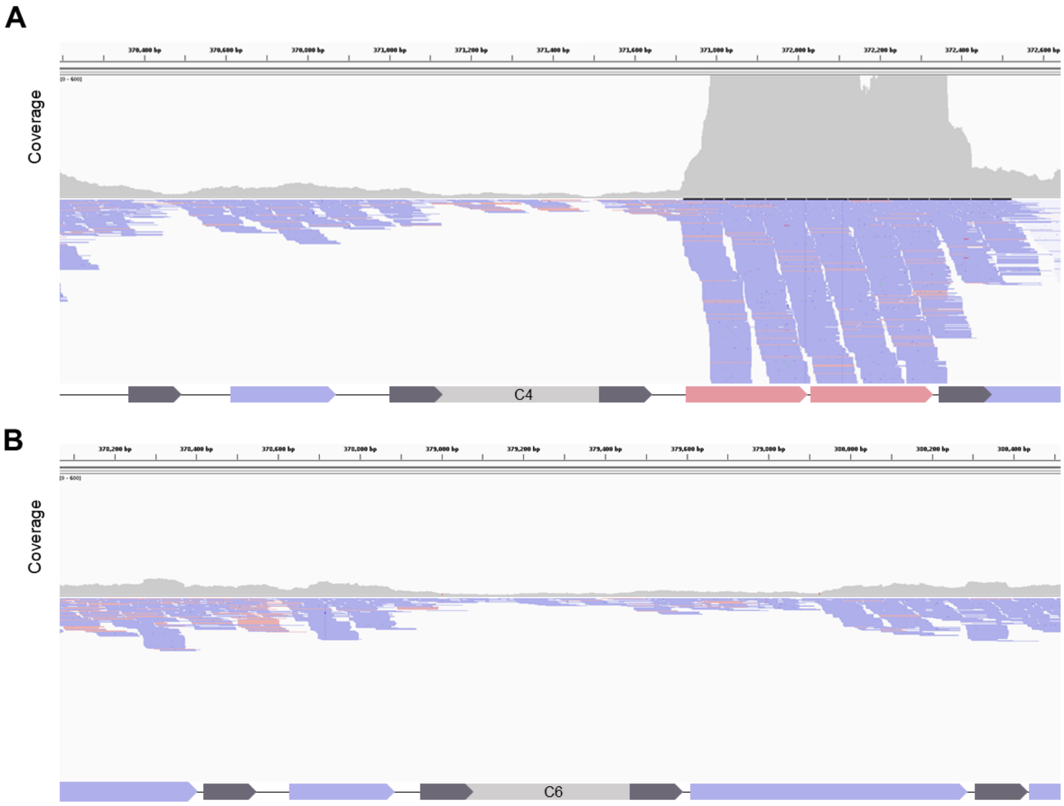

Supplementary Figure S8. Representation of reads from the RNAseq of *V. cholerae* N16961 superintegron. The number and orientation of reads, and the coverage upstream and downstream gene-less cassettes C4 (**A**) and C6 (**B**) were analyzed by Integrative Genomics Viewer (IGV) software. Blue color designates genes and reads from the plus (sense) strand; red color designates genes and reads from the minus (antisense) strand.
